# Supplementary figures and images for: Mitophagosomes induced during EV-D68 infection promote viral nonlytic release
Source: bioRxiv. 2024 Dec 6:2024.12.05.627125. Preprint. [Version 1] doi: 10.1101/2024.12.05.627125 (PMC11643070; doi:10.1101/2024.12.05.627125)

## Supplemental Figure 1. EV-D68 cleaves mitofusin-2 in A549 cells

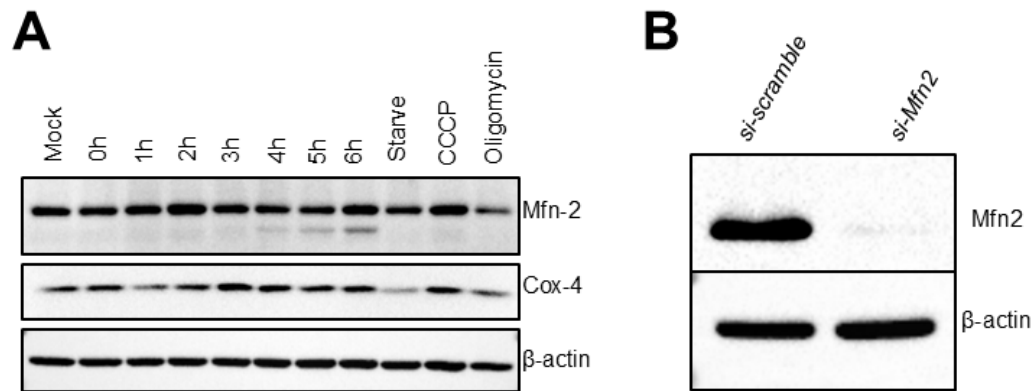

Supplement: Supplement 1 [file NIHPP2024.12.05.627125v1-supplement-1.pdf]
